# Light, Matter, Action: Shining Light on Active Matter

**DOI:** 10.1021/acsphotonics.3c00140

**Published:** 2023-04-17

**Authors:** Marcel Rey, Giovanni Volpe, Giorgio Volpe

**Affiliations:** † Physics Department, University of Gothenburg, 41296 Gothenburg, Sweden; ‡ Department of Chemistry, University College London, 20 Gordon Street, WC1H 0AJ London, United Kingdom

**Keywords:** active matter, light, intensity, wavelength, polarization, momentum transfer

## Abstract

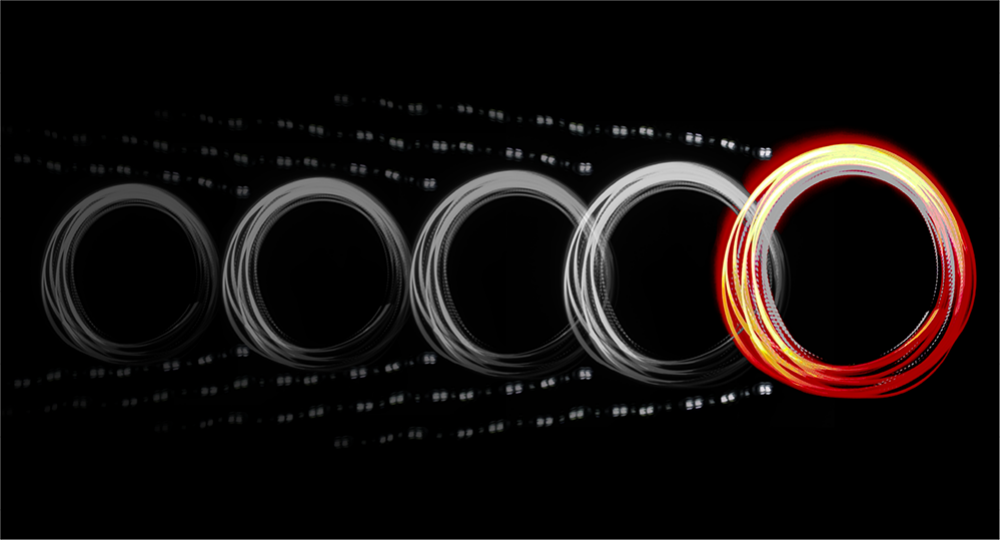

Light carries energy and momentum. It can therefore alter the motion of objects on the atomic to astronomical scales. Being widely available, readily controllable, and broadly biocompatible, light is also an ideal tool to propel microscopic particles, drive them out of thermodynamic equilibrium, and make them active. Thus, light-driven particles have become a recent focus of research in the field of soft active matter. In this Perspective, we discuss recent advances in the control of soft active matter with light, which has mainly been achieved using light intensity. We also highlight some first attempts to utilize light’s additional properties, such as its wavelength, polarization, and momentum. We then argue that fully exploiting light with all of its properties will play a critical role in increasing the level of control over the actuation of active matter as well as the flow of light itself through it. This enabling step will advance the design of soft active matter systems, their functionalities, and their transfer toward technological applications.

## Introduction

In the last half century, the possibility of transporting and actuating objects with light has left the realm of science fiction to impact several fields of science and technology. Nowadays, a tremendous number of disciplines and applications benefit from actuating objects with light. These include optical manipulation,^1^ microfluidics,^2^ nanomedicine,^3^ manufacturing,^4^ and even space exploration.^5,6^

Light carries energy and momentum that can be transferred to materials via different types of light–matter interactions.^7^ While these effects are usually too small to be appreciated in our everyday life, their magnitude is big enough to influence the motion of microscopic objects,^8^ whose energy fluctuations are comparable to the characteristic thermal energy *k*_B_*T*, with *k*_B_ being the Boltzmann constant and *T* being the absolute temperature (*k*_B_*T* ≈ 4.14 × 10^–21^ J at room temperature). This is the realm of soft matter, i.e., the branch of science that studies systems and materials that can be deformed by relatively low energies on the order of thermal fluctuations. Since the characteristic energy of a visible light photon is comparable to *k*_B_*T*, light is particularly well suited to interact with soft materials (e.g., a green photon has energy *E*_photon_ = *hc*/λ ≈ 3.8 × 10^–19^ J ≈ 90 *k*_B_*T*, where *h* is the Planck constant, *c* is the speed of light, and λ = 532 nm is the wavelength).

Active matter is a term used to include all *living* and *artificial* systems that can autonomously perform work for different tasks (e.g., move, transport cargo, and energy conversion) by utilizing the energy available to them in their environment. These systems can develop rich forms of self-organization and collective dynamics,^9^ leading to the emergence of complex properties, such as the possibility of interacting and evolving autonomously.^10−12^ At the macroscopic scale, examples of living active matter include animal groups and human crowds,^9^ while active granular matter^10^ and robotic swarms^13^ represent their artificial counterparts. At the microscale (the focus of this Perspective), examples of living active matter include bacterial cells^14^ and sperm cells,^15^ while self-propelling colloids and microrobots are their man-made analogues.^16^ Systems at this scale are characterized by two main features: (1) Brownian fluctuations can influence these systems’ motion, and (2) inertia can be neglected.^17^

**Figure 1:**
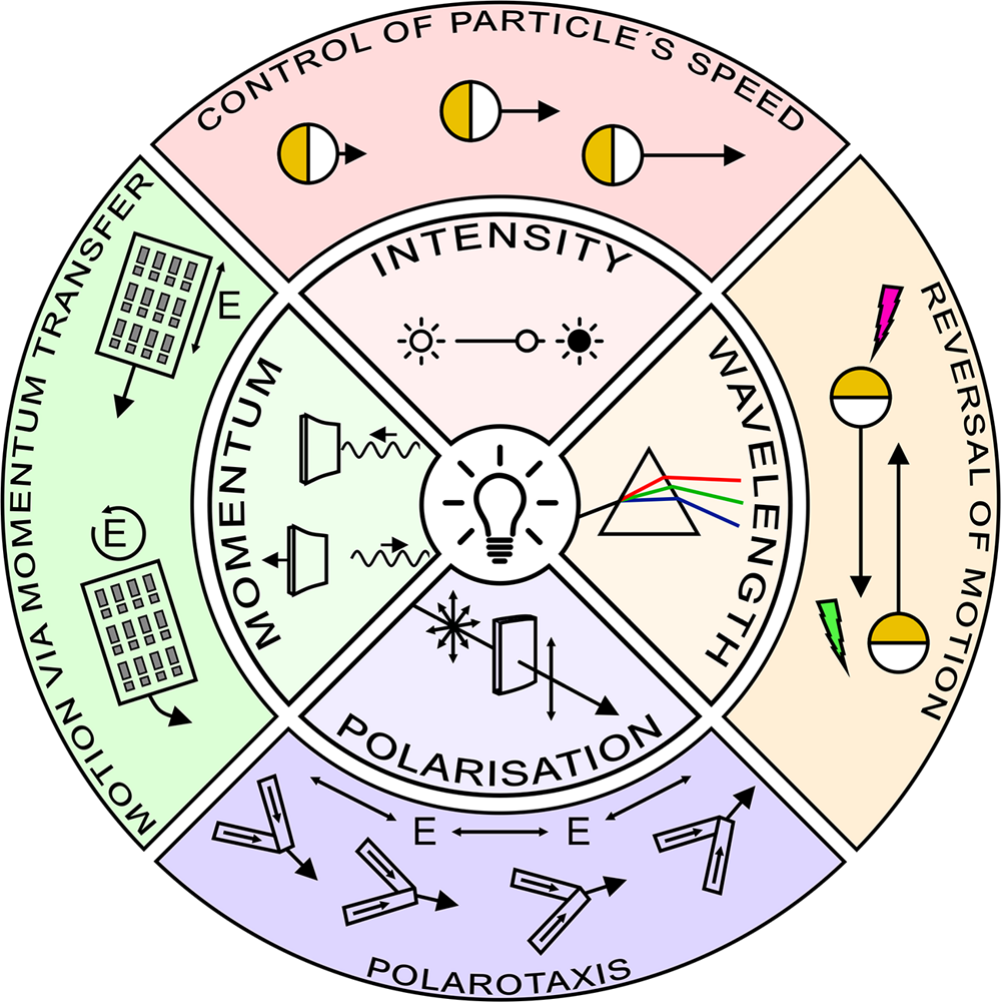
Actuation of active matter by different properties of light. Different properties of light (intensity, wavelength, polarization, and momentum) can be employed to control active matter. These schematics represent the main properties of light (inner circle) and some prominent examples of actuation (outer circle). (Top) Intensity of light typically correlates with the magnitude of the respective particle’s propulsion mechanism,^18^ so that the speed of the active particles can be adjusted by intensity. (Right) Different wavelengths can address different parts of heterogeneous active Janus particles, enabling control over propulsion direction and magnitude.^19^ (Bottom) Nanomotors consisting of nanowires with a high dichroic ratio preferentially absorb polarized light, enabling polarotactic active movement controlled by the polarization state of the incident light.^20^ (Left) Light also carries momentum, which can propel matter: for example, microvehicles bearing metasurfaces that scatter light directionally can be accelerated via transfer of light momentum.^21^

Being widely available, readily controllable, and broadly biocompatible, light is an ideal tool to control microscopic particles and drive them out of thermodynamic equilibrium, thus making them active (Figure 1). While the active matter community has enthusiastically adopted this tool to control microscopic active particles (e.g., bacteria, active colloids, and active droplets), most studies have focused on exploiting the intensity of light, while neglecting the other properties offered by light, such as its wavelength, its polarization, and its linear and angular momenta (Figure 1). Higher control will enable more fundamental scientific discoveries about far-from-equilibrium phenomena,^16^ while being useful for applications, e.g., in sensing, nanomedicine, and materials science.^22^ Nowadays, the prospects for light actuation are ever brighter thanks to the development of several new technologies, such as cheaper lasers at all wavelengths, more versatile spatial light modulators, higher-speed cameras, and advanced particle tracking algorithms based on machine learning.^23−26^

In this Perspective, we first discuss recent advances in the control of soft active matter actuation with light using its simplest property (light intensity), with a focus on microscopic active systems such as microorganisms, active colloids, and droplets. We then highlight first attempts and potential future mechanisms to utilize light’s additional properties, such as its wavelength, polarization, and momentum. Finally, we propose potential avenues to increase the level of control over the actuation of soft active matter based on fully exploiting light’s properties.

## Active Matter Actuation by Light Intensity

**Figure 2:**
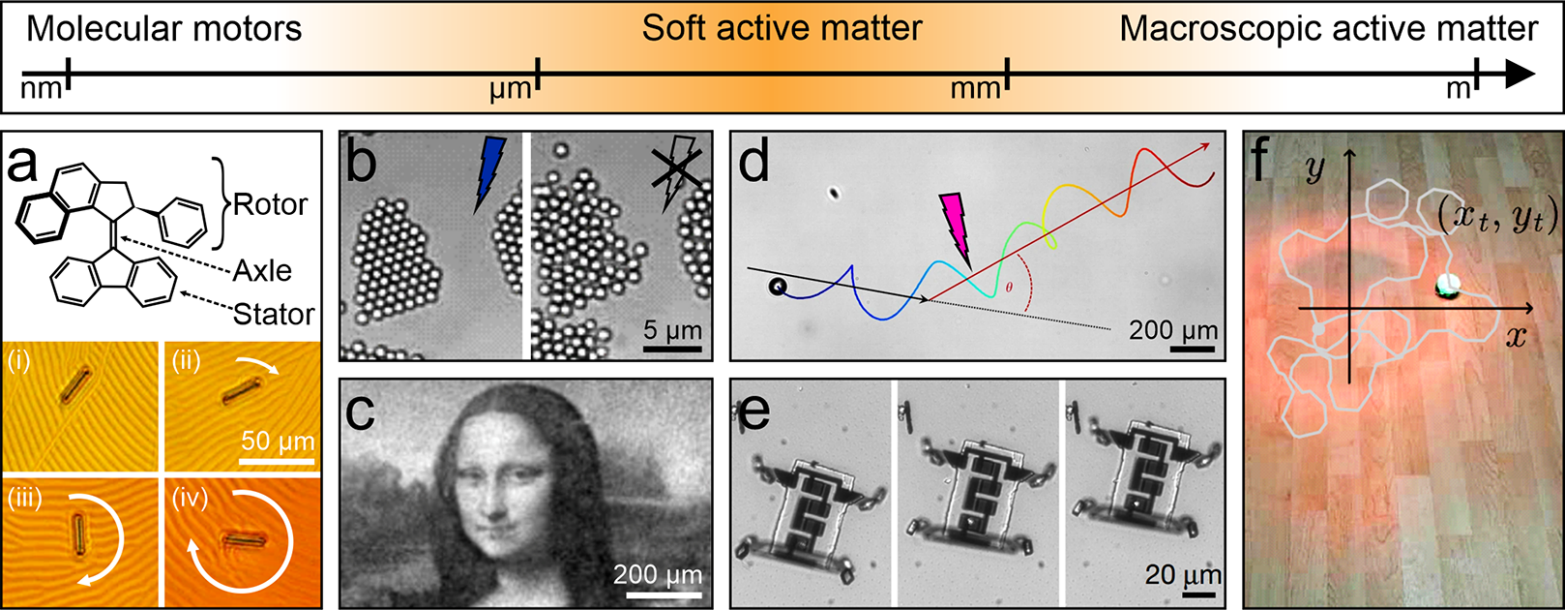
Active matter systems controlled by light intensity. Light intensity has been employed to control active matter systems at all length scales. (a) On the molecular scale, light-responsive nanomotors can undergo a photochemical isomerization around the central double bond upon irradiation with UV light that results in helicity inversion (from right-handed to left-handed).^35^ This motor is very effective at inducing helical organization in a liquid-crystal film, which can be harnessed to move microparticles placed on top of it (i–iv). Adapted with permission from ref (35). Copyright 2006 Springer Nature. (b–e) Microscopic systems. (b) Light-activated Janus colloids self-organize into clusters under blue light but dissolve when the light source is turned off. Adapted with permission from ref (29). Copyright 2013 American Association for the Advancement of Science. (c) Bacteria, genetically modified to swim smoothly with a light-controllable speed, can be arranged into complex and reconfigurable density patterns such as a portrait of Mona Lisa using a simple digital light projector. Adapted with permission under a Creative Commons CC-BY 4.0 License from ref (30). Copyright 2018 eLife Sciences Publications Ltd. (d) Self-propelling droplets of chiral nematic liquid crystals in surfactant-rich water propel in a screw-like motion. Photoinvertible chiral dopants allow conversion between right-handed and left-handed trajectories upon UV irradiation. Adapted with permission under a Creative Commons CC-BY 4.0 License from ref (31). Copyright 2019 Springer Nature. (e) Electronically integrated micromotors consisting of a body containing standard silicon electronics and surface electrochemical actuator legs are able to walk by directing laser light to its photovoltaics that alternately bias the front and back legs. Adapted with permission from ref (36). Copyright 2020 Springer Nature. (f) On the macroscale, phototactic robots can respond to light gradients, e.g., by adjusting their speed in response to the measured light intensity. Adapted with permission under a Creative Commons CC-BY 3.0 License from ref (34). Copyright 2016 Springer Nature.

Thanks to the technological developments in recent decades, highly controllable lasers and other light sources are nowadays easily available to most research laboratories. Light intensity is the most obvious light property that can be exploited to enable control over the actuation of microscopic active matter. This has been done at various scales, from molecular motors^27,28^ (Figure 2a) to microscopic colloids^29^ (Figure 2b), bacteria^30^ (Figure 2c), and droplets^16,31^ (Figure 2d), and to microrobots^32,33^ (Figure 2e) and macroscopic robots^34^ (Figure 2f). In the following, we analyze how light intensity has been used to control the behavior of these systems, with an emphasis on systems at the micrometer scale (Figure 2b–e).

### Microorganisms

Several microorganisms, including archea, bacteria, and protists, have evolved to sense and respond to light. Phototaxis, whether toward (positive) or away from (negative) a light source, can be advantageous to optimize biological and physiological functions, such as photosynthesis, growth, and the uptake of resources in competitive ecological contexts. For example, positive phototaxis can be beneficial for phototropic microorganisms, helping them to position and orient themselves to efficiently perform photosynthesis.^37^ In these microorganisms, the response to light is usually mediated by photoreceptor proteins (e.g., proteorhodopsin and rhodopsin pigments) that are sensitive to light.^37^ Most of these microorganisms can measure light intensity gradients and move accordingly performing a biased random walk toward higher or lower light intensities. This is either achieved by probing changes in the signal over time^38^ or, in more complex microorganisms, by directly measuring the gradient direction.^39,40^ A beautiful example of the latter is represented by the unicellular cyanobacterium *Synechocystis* that can accurately and directly sense the position of a light source as the cell itself acts as a spherical microlens, allowing it to see the source and move toward it.^40^ The interaction among multiple phototactic microorganisms can lead to the emergence of collective phenomena. For example, bioconvective flows form in systems of phototactic algae due to the formation of uneven mass distributions of the cells moving toward a light source.^41,42^ Time and space variations of the source lead to the dynamic triggering and reconfiguration of these bioconvective plumes.^41,42^

Beyond naturally photoresponsive microorganisms, the advent of optogenetics has enabled researchers to introduce exogenous DNA into non-photosensitive cells to express the production of light-sensitive proteins.^43^ For example, scientists have engineered *Escherichia coli* bacterial cells to respond to red, green, and blue light with the production of different pigments creating color photographs.^44^ Light-sensitive proteins have been also expressed in *E. coli* to modulate their motility and consequently their population density by light,^45^ permitting the generation of dynamic bacterial patterns and images (Figure 2c).^30,46^

### Micromotors

Inspired by these phototactic microorganisms, various man-made self-propelling microscopic particles that can move in response to light have been developed (see, e.g., recent reviews^22,47−53^). In a homogeneous light field, their motion can be described as a persistent random walk, similar to motile microorganisms in homogeneous environments. In the presence of a light intensity gradient, their motion can become biased.^16^ A paradigmatic example of micromotors is constituted by Janus particles (named after the two-faced Roman god).^54^ These are colloidal particles whose surface presents two different physicochemical properties.^55,56^ This asymmetry induces a local gradient in some thermodynamic properties (e.g., concentration, interfacial energy, or temperature) across the particle that leads to its self-propulsion.^16,54^ Light can be used to create such an asymmetry across an illuminated particle (e.g., in its temperature profile or surface chemistry). One approach relies on coating one side of the particle with a photocatalytic material (such as platinum, palladium, hematite, or titania) to locally decompose a chemical fuel (usually hydrogen peroxide) in water and create a local concentration to drive the particle’s self-diffusiophoresis^29,57−59^ or self-electrophoresis.^60−62^ Alternatively, light absorption in Janus particles half-coated with a light-absorbing material (e.g., gold or carbon) can also lead to self-propulsion directly or indirectly due to the formation of a local temperature gradient across the particle because of selective heating at the absorbing side.^18,56,63−67^ UV light can also tune the mobility of electrically powered semiconductor-dielectric Janus particles by increasing the contrast in the polarizability between the two materials, which results in an increased electrokinetic mobility.^68^ Differently from their biological counterparts which can move by body deformation, most of these synthetic micromotors have rigid shapes and only recently have light-responsive reconfigurable microswimmers been proposed.^69−73^ As in the case of microorganisms, artificial Janus particles can also orient in the light field and feature a biased directional phototactic behavior in a light intensity gradient.^18,74,75^

While at the individual particle level these artificial micromotors have found interest as a promising route to develop novel applications in nanomedicine and environmental remediation,^49,51^ complex collective behaviors have also been reported when these individual units self-organize into larger light-activated clusters, including the formation of living crystals (Figure 2b),^29^ inverse crystallization,^76^ crystal annealing,^77^ different lattice structures,^78^ active colloidal molecules,^79−81^ dynamic pattern formation,^82−84^ metamachines,^85^ and even functional photonic materials.^86^ These emerging behaviors are interesting as model systems to study self-organization in living matter and as a novel route to develop next-generation materials. Mostly, these complex collective behaviors are governed by physical forces such as steric, phoretic, and hydrodynamic interactions. Recently, however, light has been used to encode predefined feedback interactions among the active particles (e.g., attractive, repulsive or aligning interactions based on the knowledge of the position of nearby particles).^87−89^ The introduction of these interactions has enabled more complex programmable active matter behaviors, which include dynamic active particle molecules, visual-perception-dependent motility, and reinforced learning.^87−89^

### Active Droplets

Droplets are small volumes of liquid separated from their surroundings by at least one interface.^90^ Because of their small size, they can be used as versatile transport vessels and reactors in microfluidics for applications in chemistry, biology, and nanomedicine.^90^ Active droplets are a particular class of droplets that can either self-propel in isolation or do so in response to other neighboring droplets in an emulsion.^91,92^ The main physical effect that induces these droplets’ motion is the Marangoni effect, where a gradient of surface tension drives mass transport toward areas of higher surface tension. In the case of active droplets, the Marangoni effect is (self-) induced by the droplet itself or by surrounding droplets. Such gradients of surface energy can be produced optically, e.g., by illuminating the droplet surface and harnessing the thermal or photochemical effects of the light absorbed within the droplet.^2,92−95^ For example, lipophilic droplets stabilized by photoresponsive surfactants can move in light gradients. Light irradiation induces the dissociation of photoresponsive surfactants combined with a rapid pH change in the surrounding aqueous phase, which results in fast movement of the droplet away from the light source due to a change in surface tension.^96^ In self-propelling liquid-crystal droplets containing photoinvertible chiral dopants, light irradiation allows converting between right-handed or left-handed screw-like trajectories (Figure 2d).^31^ Recently, similar light-induced motility effects away or toward a light source were also reported in droplets of various materials with the addition of different photoresponsive molecules, such as surfactants^97,98^ and inorganic particles.^99^

### Microrobots

The possibility of fabricating active particles with complex functionalities has driven the development of novel microrobots, i.e., robots with characteristic sizes below 1 mm.^32,100^ While these systems are at the very edge of what is typically considered soft active matter, they are a powerful reminder of what can be achieved by light actuation. Due to their relatively larger size, inertial effects, rather than viscous, are more prominent in determining their motion, and Brownian fluctuations less relevant.^101^ In particular, liquid-crystalline elastomers (LCE) are a common material employed to realize biomimetic micromotors capable of autonomous locomotion in response to light.^102^ Beyond the realization of devices at the millimeter scale,^103−107^ these materials have been successfully employed to realize microrobots in the sub-millimeter range, such as walkers on solid surfaces^108^ and biomimetic swimmers in fluids.^109^ Similar shape-changing walkers and grippers were realized based on photosensitive spiropyran-based hydrogels.^110,111^ Recently, a new class of microrobots has been demonstrated that integrated electronic components with light actuation, thus providing a stepping stone toward mass-manufacturing silicon-based functional robots at the microscopic scale (Figure 2e).^36^

## Actuation by Other Properties of Light

Differently from intensity, other light properties have not yet been extensively exploited for the actuation of soft active matter. In this section, we will briefly review how they have been used so far in this context (with a focus on wavelength, polarization, and transfer of momentum) and what further possibilities they offer.

### Wavelength

The wavelength determines the color of the light as well as the energy carried by each photon. Optimal light conditions are crucial for microorganism, e.g. cyanobacteria,^112^ that grow by capturing energy from sunlight. They demonstrate positive phototaxis toward green light as it is their preferred energy source for oxygenic photosynthesis, while they show negative phototaxis away from strong light or UV light as it causes cell damage.^112^ Similarly, marine zooplankton *Platynereis* larvae exposed to UV light swim downward, away from the light source, while cyan light makes the larvae swim in the reverse, upward direction.^113^ In the ocean, UV light is most intense near the surface, while cyan light reaches greater depths. *Platynereis* larvae may thus use the ratio between UV and cyan light as a “depth gauge” during vertical migration.^113^

In artificial active matter, wavelength is the second most important and exploited property of light after intensity. The wavelength is often a boundary condition imposed by the materials employed in the experiment. For example, Janus particles with metallic caps can be heated by light of a specific wavelength depending on the cap’s material, e.g., green matches the plasmon resonance of gold (λ ≈ 530 nm), blue that of silver (λ ≈ 400 nm), and UV that of platinum (λ ≈ 260 nm).

**Figure 3:**
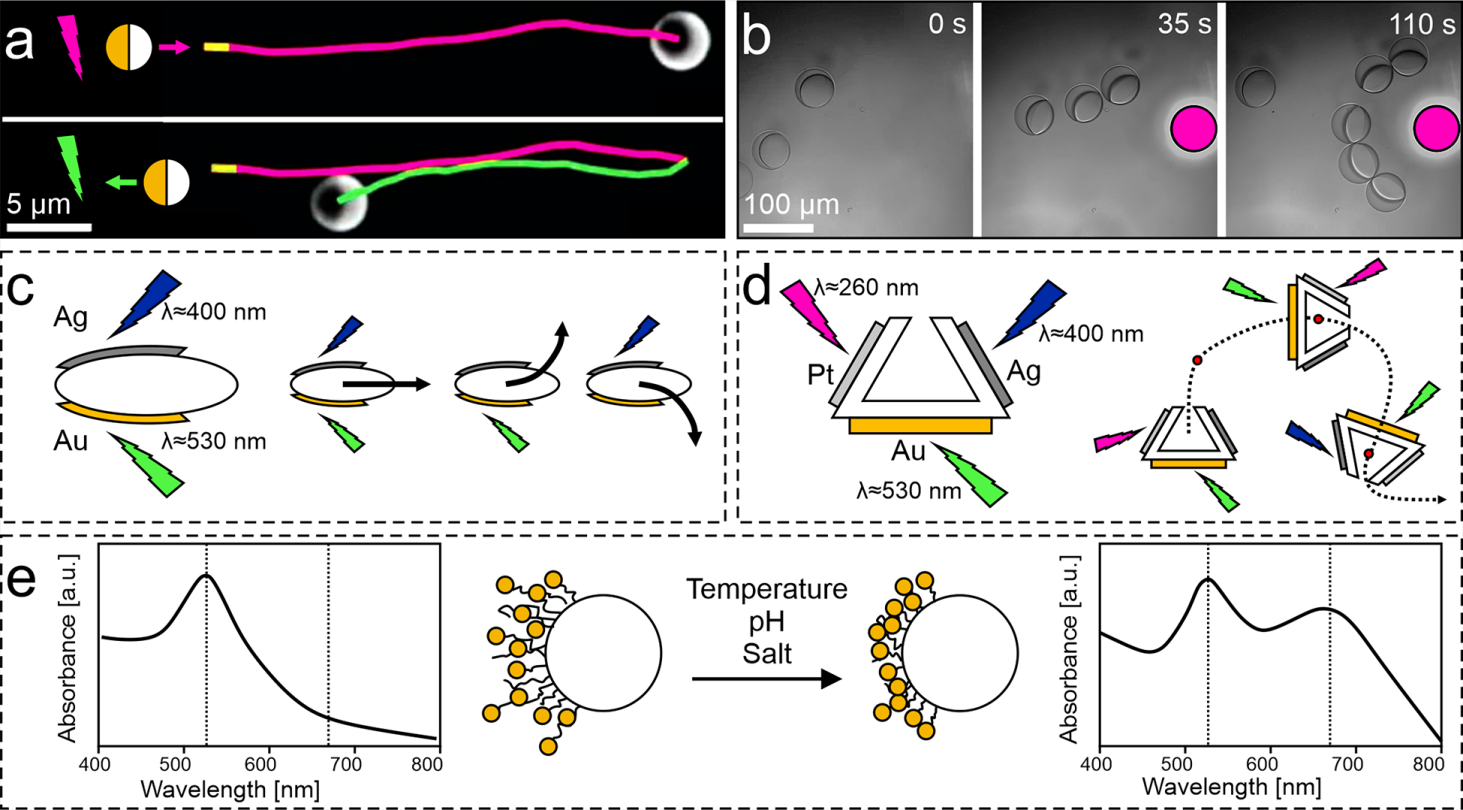
Active matter systems controlled by wavelength. (a) Forward trajectory of Au-coated anatase TiO_2_ Janus particles in a H_2_O_2_ solution upon illumination with UV light (top, magenta trajectory) and reverse direction upon illumination with green light (bottom, green trajectory). Adapted with permission under a Creative Commons CC-BY 4.0 License from ref (19). Copyright 2020 Springer Nature. (b) AzoTAB-stabilized Janus emulsions under bright-field blue light irradiation self-assemble toward a localized UV light spot (filled circle). Adapted with permission under a Creative Commons CC-BY 4.0 License from ref (114). Copyright 2022 Springer Nature. (c–e) Potential future uses of wavelength as control strategy for active particles. (c) Elongated particles (e.g., ellipsoids) with two different metal patches can be steered using two wavelengths of light. (d) U-shaped particle with three metal patches can work as cargo carriers, with full control over their planar movement. (e) In Janus particles coated with stimuli-responsive polymer brushes decorated with plasmonic nanoparticles, external stimuli such as temperature, pH, or salt concentration can collapse the polymer and shift the absorbance spectrum,^115^ affecting the particle mobility.

Multiple wavelengths have been combined in a single experiment to control the propulsion of different types of active particles^116^ (or different parts of an active particle) to achieve more complex particle’s behaviors.^19,117−119^ For example, TiO_2_ Janus particles with either cobalt oxide caps^119^ or metal caps^19,117,118^ combine a complex interplay between the adsorption of light at different wavelengths and the respective catalytic and photochemical processes occurring on each side of the particle. Adjusting the wavelength of light enables control over the propulsion direction (including its on-demand reversal)^19,117^ and magnitude^19,117−119^ (Figure 3a). Similarly, hybrid active particles made from two different photocatalysts, e.g., TiO_2_ and CuO_2_, catalyze hydrogen peroxide over differing ranges of wavelength,^120^ which can lead to a wavelength-dependent translational and rotational swimming behavior.^120^ Alternatively, photoelectrochemically driven nanotree microswimmers loaded with photosensitizer dyes can be driven and steered by visible light using different wavelengths.^116^

Strategies to manipulate liquid interfaces and droplets typically employ azobenzene-derived surfactants such as AzoTAB.^121^ These molecules can be reversibly switched between two conformations of different polarity by subsequent illumination with UV (365 nm) and blue (475 nm) light, which affects the interfacial tension of interfaces stabilized by the surfactant. For example, this principle enabled the omnidirectional manipulation of oil droplets^122^ and liquid marbles^123^ floating on the surface of an aqueous solution of AzoTAB surfactants. By changing the wavelength of the light used to illuminate the edge of the droplets or liquid marbles, it was possible to reversibly repel them from the incident beam (UV illumination) or attract them toward it (blue illumination).^122,123^ Similarly, oil droplets can be propelled in the proximity of azobenzene-stabilized micelles.^124^ Changing the wavelength of the light induces a change in the micelle geometry, which impacts the movement pattern of the droplets, resulting in a run-and-halt behavior.^124^ Further, Janus emulsions stabilized with AzoTAB under blue light irradiation move toward a UV light spot around which they self-assemble in an ordered fashion (Figure 3b).^114^ Finally, fatty acid droplets containing photosensitive spiropyran can move toward visible light sources and away from UV light sources, thus enabling their manipulation in three dimensions.^125^

Potential future uses of the wavelength as a control mechanism of an active system may include:

- **Multiple resonant shapes**. Complex active particle shapes can be designed to respond to different wavelengths, e.g., by exploiting characteristic plasmonic resonances (hence wavelength-selective enhanced light absorption) of different metals. For example, ellipsoidal or rod-shaped anisotropic Janus particles with two different metal patches instead of one^126^ would enable addressing each individual patch or both simultaneously by adjusting the wavelength of light. The anisotropic nature of the particle should allow control over the direction and magnitude of propulsion, where particles can either move straight or rotate clockwise or counterclockwis depending on the light wavelength and intensity (Figure 3c). The same underlying principle can be used to design even more complex units, e.g., U-shaped particles with multiple plasmonic patches, which could additionally allow the reversal of the direction of motion for loading/unloading cargoes (Figure 3d).
- **Stimuli-responsive plasmonic or photonic resonance shifts via elastic deformations**. The elastic deformation of stimuli-responsive polymer brushes decorated with plasmonic nanoparticles has been employed to tune the wavelength of the light absorbed by this composite structure: external stimuli such as changes in pH,^127^ temperature,^128^ or salt concentration^115^ can collapse the polymer brush and bring the plasmonic nanoparticles in closer contact, thus red-shifting the absorption spectrum. We suggest to employ the same concept for active particles, which will then be able to adapt their activity to their local environment (Figure 3e). A further, though slightly more futuristic, approach is inspired by the skin of chameleons and cephalopods: these animals can camouflage by actively tuning the photonic response of their skin through elastic deformation, thus changing the wavelength of the reflected and absorbed light. Similarly, photonic active particles could be realized with stimuli-responsive hydrogel opal films,^129^ where the photonic band gap can be adjusted via external stimuli such as temperature.

### Polarization

Polarization is the property of light waves, which describes the oscillation of the electric field in the direction perpendicular to the wave propagation. For example, light from the sun is unpolarized, i.e., there is no preferred orientation for this oscillation. Unpolarized light can become polarized when it is scattered or passes through polarizing filters that select only certain orientations of the electric field. In linearly polarized light, the electric field oscillates in a single direction perpendicular to the propagation direction. In circularly polarized light, the field rotates at a constant rate around the direction of propagation, as the wave travels.

**Figure 4:**
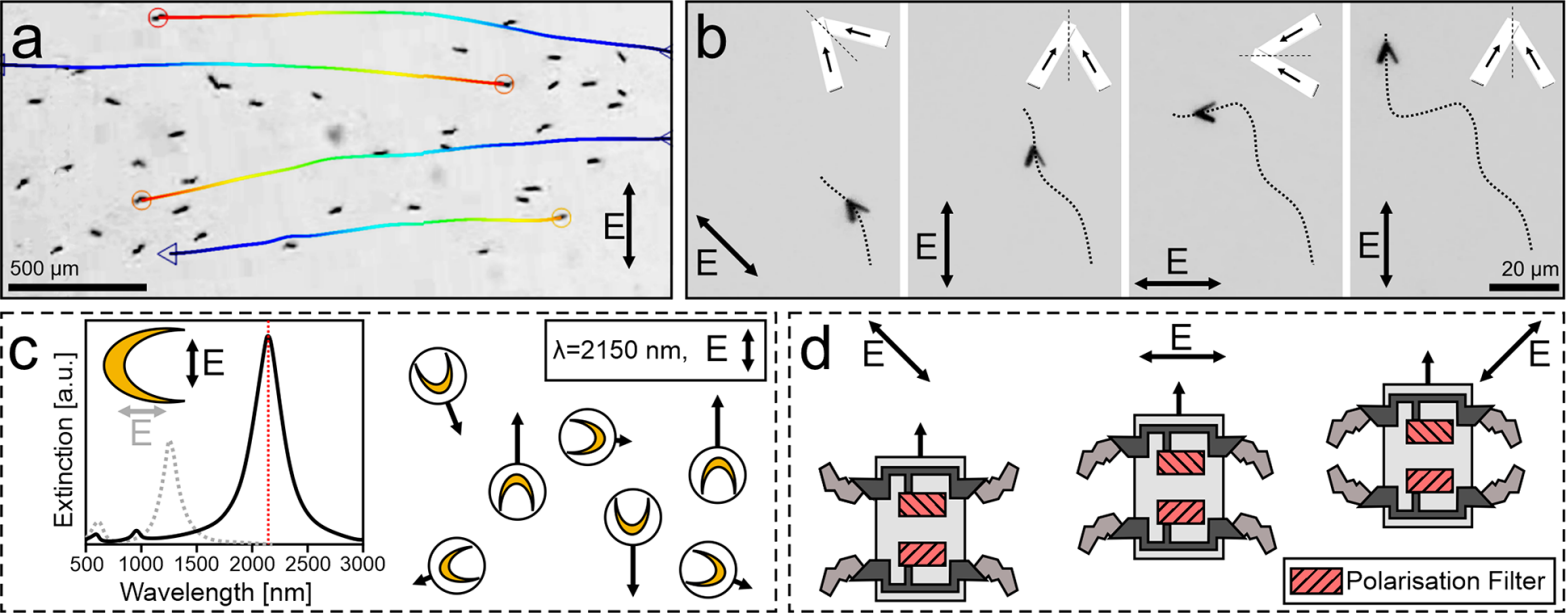
Active matter systems controlled by polarization. (a) Polarotaxis in photoresponsive algae (*Euglena gracilis*) leads to movement perpendicular to the polarization of light. Adapted with permission from ref (134). Copyright 2021 American Physical Society. (b) Nanomotors consisting of nanowires with a high dichroic ratio preferentially absorb polarized light, enabling polarotactic active movement and steering controlled by the polarization state of the incident light. Adapted with permission from ref (20). Copyright 2019 John Wiley and Sons. (c,d) Potential future uses of polarization as control mechanism in active particles. (c) Plasmonic nanocrescents feature polarization-dependent resonances and near-field enhancement at their tips.^135^ For a fixed wavelength, the propulsion strength of active particles driven by similar nanostructures therefore would depend on their orientation relative to the polarization of light, leading to predominant motion in the direction where the absorption of a given polarization is stronger. (d) Electronically integrated micromotors^36^ equipped with polarizing filters in front of photovoltaic components could allow the polarization-dependent control of specific actuators to steer the particle’s self-propulsion with the light polarization.

Sensitivity to polarization is not uncommon in nature. For example, many insect species bear photoreceptors in a small dorsal rim area of the eye that detect polarized skylights to improve their navigation skills.^130^ Furthermore, polarization sensitivity helps squids detect transparent yet polarization-active zooplankton under partly polarized light.^131^ The vision of the mantis shrimp holds the world record for the most complex visual system: these marine crustaceans have up to 16 photoreceptors and can see UV, visible, and polarized light; they are also the only animal known to detect circularly polarized light, which may serve as a secret communication system.^132^ At the microscale, *Euglena gracilis* cells (alga) exhibit polarotaxis behavior, which aligns their motion direction perpendicular to the light polarization.^133^ Their polarotaxis can also be used to guide the collective movement of these algae (Figure 4a).^134^

In the man-made world, polarized light has been widely exploited in optical manipulation to control the orientation and rotation of particles via transfer of linear and spin angular momentum.^7^ However, only recently have nanomotors with a high dichroic ratio been shown to respond to polarized light. These nanomotors are constituted of nanowires with a ZnO shell and a Sb_2_Se_3_ core, whose anisotropic crystal structure preferentially absorbs light polarized along the wire, hence enhancing their self-propulsion speed (Figure 4b).^20^ By connecting two cross-aligned dichroic nanowires, the authors of this work were able to realize artificial polarotactic active particles whose navigation can be controlled by the polarization state of the incident light.^20^

However, when it comes to actuating artificial active matter, polarization is still under-explored. Examples of possible future uses of polarization to control the motion of active particles include the following:

- **Polarization-dependent absorbers**. The scope for active particles whose self-phoretic forces depend on the absorption of specific polarizations of light is broad. This can be achieved employing dielectric structures (Figure 4b)^20^ or metallic structures, e.g., the plasmonic nanocrescents in Figure 4c, which feature multiple polarization-dependent resonances combined with near-field enhancement at their tips.^136,137^ The propulsion speed of active particles featuring similar nanostructures would therefore depend on their orientation relative to the polarization of light. For example, in Figure 4c, this would lead to an enhanced propulsion of the particles in the direction parallel to the polarization of light, thus leading to a polarotactic behavior as the algae cells in Figure 4a.^134^
- **Polarized photovoltaics**. The integration of electronic components in microrobots^36^ and metavehicles^21^ could be exploited to generate polarization-dependent motion. The electronic components could be simple circuits made from standard inorganic or organic photovoltaics and metal interconnects powering some actuators on the active particle.^36^ The use of polarizing filters in front of the photovoltaic components could allow the polarization-dependent control of specific actuators to steer the particle’s self-propulsion with the light polarization (Figure 4d).

### Transfer of Momentum

The existence of light momentum is foundational to the whole field of optical trapping and optical micromanipulation.^7,8,138^ Light can carry two kinds of angular momenta: spin angular momentum and orbital angular momentum. Spin angular momentum depends on the polarization state of the light: it is zero for linearly polarized light and maximum for circularly polarized light (but with opposite signs for left and right polarization). Orbital angular momentum is typically associated with structured light beams, such as Laguerre–Gaussian beams, that carry a topological charge. When light interacts with matter, the changes in linear and angular optical momentum induce forces and torques. In most cases, these forces and torques are used to hold particles in place or to generate some deterministic, controllable motion. There are nevertheless some cases in which they have been employed to alter the random motion of microscopic particles in interesting ways. For example, the forces produced by random light fields have been employed to alter the diffusion of Brownian particles^139−145^ leading also to superdiffusive behavior in the presence of time-varying patterns.^140,146,147^

**Figure 5:**
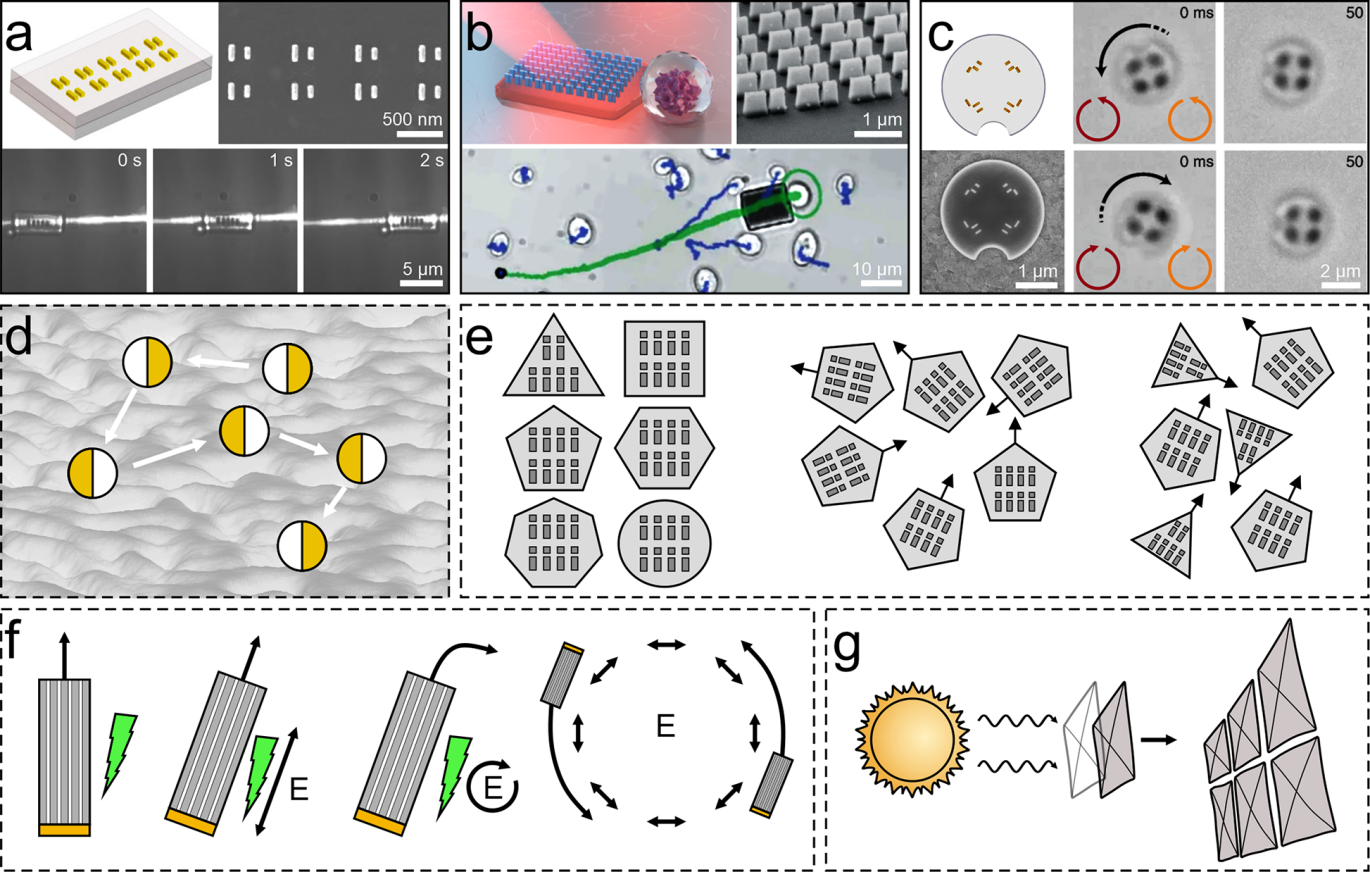
Active matter systems controlled by transfer of light momentum. (a) Plasmonic linear nanomotor driven by momentum transfer of light. Adapted with permission under a Creative Commons CC-BY 4.0 License from ref (148). Copyright 2020 American Association for the Advancement of Science. (b) Microvehicles containing a directional scattering metasurface. The transfer of momentum leads to straight propulsion under linearly polarized light and circular motion under circularly polarized light. These metavehicles can also be used to move some microorganisms present in the solution (bottom panel). Adapted with permission from ref (21). Copyright 2021 Springer Nature. (c) Light-driven microparticles containing four chiral plasmonic resonators are maneuvered by adjusting the optical power for each resonator using two overlapping unfocused light fields at 830 nm (orange arrow) and 980 nm (red arrow) with right- and left-handed circular polarization, respectively. Adapted with permission from ref (149). Copyright 2022 Springer Nature. (d–g) Potential future uses of momentum transfer in active matter. (d) Active particles either propelled by light or by chemical fuels can explore a speckle light pattern according to some nontrivial random motion statistics (e.g., by a Fickian yet non-Gaussian diffusion^142^). (e) Active momentum-driven particles with different shapes can generate emergent collective self-assembly behaviors, as already theoretically modeled.^150,151^ (f) Active birefringent Janus particles with a metal cap on one side^152^ can combine the propulsion of Janus particles with the orientation in polarized light of birefringent particles. Such particles would move along the polarization direction of linearly polarized light or show a circular motion in circularly polarized light. (g) Solar sails^5^ propelled by the light of the sun could self-assemble in space into complex devices, e.g., space telescopes.

Within the field of active matter, recent work exploited the transfer of momentum using unfocused light to propel microscopic vehicles with incorporated plasmonic or dielectric metasurfaces that generate lateral optical forces due to directional light scattering along the side of the structure (Figure 5a–c).^21,148,149^ For example, microvehicles with directional light-scattering nanostructures arranged in parallel can propel forward upon illumination with linearly polarized light due to transfer of linear momentum (Figure 5a,b),^21,148^ and they can be steered left or right by circularly polarized light thanks to transfer of spin angular momentum (Figure 5b).^21^ As an example of application, these metavehicles were also employed for the micromanipulation of colloidal particles and microorganisms (Figure 5b).^21^ Alternately, rotation under plain linearly polarized light can be achieved by arranging the scatterers in a circle (Figure 5c).^148,149^ Moreover, microvehicles with four individually addressable chiral plasmonic nanoantennas acting as nanomotor enable full motion control in two dimensions in all three independent degrees of freedom (two translational and one rotational):^149^ Similar to macroscopic drones but in two dimensions, these microvehicles are maneuvered by adjusting the optical power for each nanomotor using two overlapping unfocused light fields at λ = 830 nm and λ = 980 nm, each with right- or left-handed polarization (Figure 5c).^149^

Further possible uses of optical forces and torques for the field of active matter are the following:

- **Complex optical fields**. The use of complex light fields (e.g., random speckle light fields) is a promising way to generate nontrivial optical potentials that can influence the individual and collective motion behavior of active particles (Figure 5d). For example, the motion of non-light-driven (e.g., catalytic) active particles within random light fields may show a competition between their propulsion activity and the retardation introduced by the speckle field as a function of increasing light intensity, which has been predicted in theoretical work.^153^ On the other hand, active particles that are driven by light may be accelerated in speckle fields leading to the emergence of superdiffusive patterns.
- **Complex metavehicles**. Metavehicles propelled by momentum transfer can be fabricated in any shape without interfering with their propulsion mechanism (Figure 5e). This makes them a promising model system to study collective phase behaviors of active particles as a function of particle shape, which has recently been theoretically modeled.^150,151^
- **Birefringent active particles**. Birefringent particles, e.g., metamaterial nanoparticles,^152^ containing an absorbing cap on one side would combine the propulsion of Janus particles with the reorientation capabilities in polarized light of birefringent particles^152^ (Figure 5f). Such hybrid particles can be expected to propel parallel to the polarization of light but also feature circular motion under circularly polarized light due to transfer of spin angular momentum.
- **Force-driven collective effects**. Self-organization of multiple active particles under the action of optical forces can find fruitful applications in space exploration. For example, several solar sails^5^ might interact and self-assemble through optical binding. The idea is that the incoming light scattered by each solar sail can then exert additional optical forces and torques on other solar sails. If properly implemented, this effect can generate some feedback loops among the solar sails that can stabilize the whole ensemble. The same concept could be also exploited to produce large self-organized devices: e.g., new space telescopes with effective mirrors made by self-organized active solar sails which might be much larger than the Webb telescope (Figure 5g).

### Further Actuation by Structured Light

**Figure 6:**
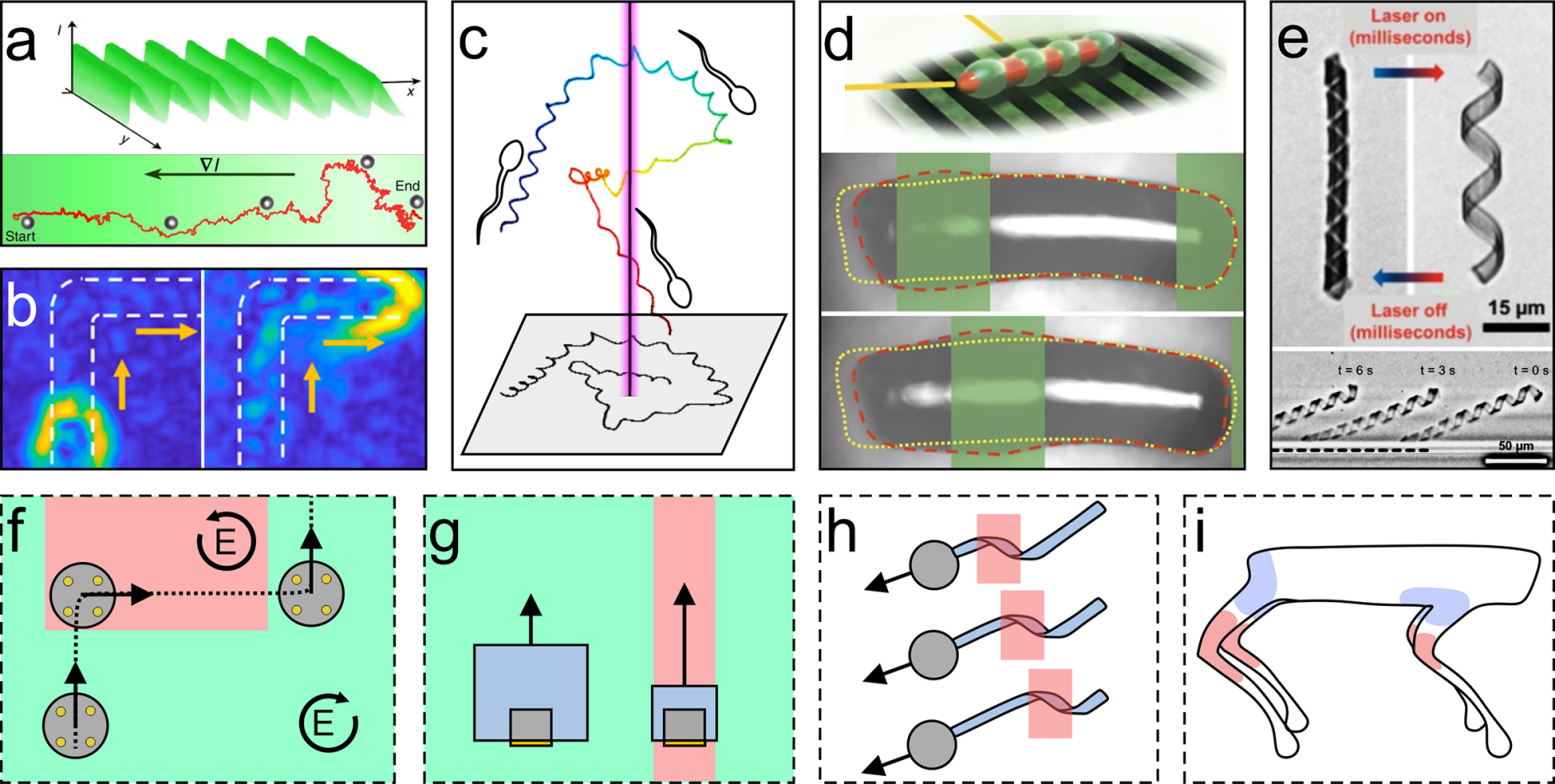
Active matter systems interacting with structured light. (a) Janus particles in a critical mixture of water–lutidine align such that they move along the gradient of light toward low light intensities. Directed particle transport over arbitrarily long distances can then be achieved using periodic sawtooth-like light profiles. Adapted with permission under a Creative Commons CC-BY 4.0 License from ref (74). Copyright 2016 Springer Nature. (b) Structured light can guide the motion waves among photochemically activated colloids. Adapted from ref (156). Copyright 2022 American Chemical Society. (c) Structured UV light can create spatiotemporal chemical landscapes by releasing caged chemo-attractants, which guide the movement of sperms. Adapted with permission under a Creative Commons CC-BY 4.0 License from ref (158). Copyright 2015 Springer Nature. (d) Spatiotemporally structured light induces intrabody shape changes in microrobots consisting of photoactive liquid-crystal elastomers, which enables self-propulsion by generating a traveling-wave motion. Adapted with permission from ref (109). Copyright 2016 Springer Nature. (e) Temporally structured light enables rapid dynamic switching between the configurations of helical composite hydrogel microrobots, enabling translational movement near a solid surface. Adapted with permission from ref (72). Copyright 2017 John Wiley and Sons. (f–i) Potential future uses of structured light to actuate and control active matter. (f) Metavehicles are able to change their motion direction (linaer or circular) depending on the polarized polarization of the illuminating light.^21,149^ Structured light landscapes with different local polarizations could guide the movement of such microvehicles. (g) Microrobots could comprise temperature-responsive bodies that shrink upon irradiation with IR light, thus reducing their drag force and increasing their propulsion magnitude. Structured light with different local wavelengths could spatiotemporally change its propulsion magnitude. (h) Spatiotemporally structured light could locally bend hydrogel nanoribbons^72,109,160^ and induce a snake-like motion, which could be exploited to propel microparticles. (i) Microwalkers could be driven by hydrogel-gold nanoparticle composites, which serve as artificial muscles and joints in response to light. Using spatiotemporally structured light, each artificial element could be addressed individually to enable microscale artificial walking.

A more holistic approach to the use of light for active matter will entail full control in space and time of its properties, including amplitude, phase, polarization, and momentum.^154,155^ Simple forms of structured light have already been employed in some active matter experiments. For example, the fact that Janus particles in a critical mixture of water–lutidine feature negative phototaxis in light gradients by drifting toward lower light intensities because of diffusiophoretic torques (Figure 6a) has been exploited in sawtooth-shaped static light profiles to make particles undergo directed motion over arbitrarily long distances (Figure 6a).^74^ Furthermore, structured light has been used to guide traveling motion waves among photochemically activated oscillating colloids.^156^ Silver chloride (AgCl) particles in dilute hydrogen peroxide solutions under UV light illumination exhibit both single-particle and collective oscillations in their motion, which arise due to an oscillatory, reversible conversion of AgCl to silver metal at the particle’s surface.^157^ These motion waves can be guided by spatial light patterns, thus enabling a precise and programmable control over the motion waves’ origin, path and direction (Figure 6b).^156^ In biological systems, structured UV light enabled the creation of defined 3D spatiotemporal chemical landscapes by releasing caged chemoattractants, which were used to investigate the chemotactic navigation mechanism of sperm (Figure 6c).^158^ Further, *E. gracilis* algae swim in polygonal trajectories when exposed to a sudden increase in light intensity.^159^ In spatially structured light landscapes with different light intensities, algae coming from low light to high light intensity start polygonal swimming or localized spinning, making the cells turn around.^159^

Structured light can also be used to induce body deformation in microswimmers, leading to their motion. For example, spatiotemporally structured light based on interference patterns was used to power and control intrabody shape changes in microrobots consisting of photoactive liquid-crystal elastomers,^109^ which were able to self-propel by generating a traveling-wave motion (Figure 6d).^109,161^ Temporally structured light was further used to actuate the soft microrobots. These microrobots consist of a temperature-responsive hydrogel filled with gold nanorods that enable fast heating/cooling dynamics upon irradiation with IR light combined with volumetric shape changes.^72^ Covering the hydrogel composite on one side with a thin gold layer restricts swelling and shrinking, leading to the formation of helical configurations.^72,160,162^ Temporally structured light enables rapid dynamic switching between left-handed and right-handed helical configurations^160^ as well as translational movement near a solid surface (Figure 6e).^72^

As the possibilities for structuring light increase with the advancement of light modulating devices, control of active matter with structured light could include the following:

- **Control of microvehicles by structured polarization**. Structured light with different local polarizations or wavelengths could guide the movement of microvehicles^21,149^ that can change their motion patters depending on the polarization of the illuminating light (Figure 6f).
- **Shrinkable microrobots in structured intensity fields**. Structured light could spatiotemporally change the propulsion magnitude of microrobots comprising temperature-responsive bodies that shrink upon illumination with infrared light,^69,72,73^ thus reducing their drag force and increasing their propulsion magnitude or propulsion direction. (Figure 6g).
- **Propulsion by deformable nanoribbons in spatiotemporally oscillating fields**. Spatiotemporally structured light could induce a snake-like motion in hydrogel nanoribbons^72,109,160^ functionalized with microparticles, which would then propel (Figure 6h).
- **3D-printed articulated microbots actuated by spatiotemporally structured light**. Recent advances in 3D printing have enabled the precise programmable control over the shape morphing and folding properties of soft stimuli-responsive composite materials.^71,163−165^ For example, embedded gold nanorods have been employed to enhance light absorption in temperature-responsive hydrogel composites.^72,160^ Printing composite hydrogels including gold nanoparticles of different sizes and aspect ratios could then enable researchers to address such hydrogels individually by exploiting different plasmonic resonances to realize artificial muscles and joints for microscale walkers and crawlers (Figure 6i).

## Light-to-Work Efficiency

Last, we compare the light-to-work conversion efficiency η = *P*_out_/*P*_in_ for different propulsion mechanisms (Table 1), where *P*_out_ = ζ*v*^2^ is the mechanical power of the active particles moving at speed *v* in a fluid with a friction coefficient ζ and *P*_in_ = *P*_d_*A* with *P*_d_ being the power density of the input light and *A* is the projected area of the active particles in the plane of motion.

**Table 1:** Comparison of Light-to-Work Efficiencies for Different Active Particles’ Propulsion Mechanisms

propulsion mechanism	particle type	efficiency	ref
transfer of momentum	microdrones	1.9 × 10^–16^	(149)
transfer of momentum	metavehicles	1.6 × 10^–15^	(21)
transfer of momentum	birefringent particles	2.6 × 10^–14^	(166)
critical demixing	Janus particles	1.9 × 10^–16^	(18)
critical demixing	Janus particles	2.3 × 10^–15^	(64)
critical demixing	Janus particles	1.2 × 10^–14^	(167)
thermophoresis	Janus particles	4.8 × 10^–16^	(63)
thermophoresis	particle-droplet dimers	6.3 × 10^–16^	(95)
thermophoresis	Janus particles	2.0 × 10^–14^	(168)
Marangoni effect	assymetric gears	3.1 × 10^–11^	(169)
Marangoni effect	Janus particles	9.5 × 10^–11^	(67)
self-electrophoresis	Janus particles	7.6 × 10^–9^	(170)
self-electrophoresis	Janus particles, H_2_O_2_	1.7 × 10^–8^	(61)
self-diffusophoresis	AgCl particles	1.2 × 10^–9^	(57)

For particles propelled via transfer of momentum, we find a comparably low efficiency of ≈10^–14^ to 10^–16^.^21,149,166^ The difference between the various mechanisms is a consequence of the number and strength of the different directional light-scattering nanostructures present per active particle. The efficiency of critical demixing varies by 2 orders of magnitude from ≈10^–16^ to 10^–14^, which is likely related to the proximity of the initial experimental temperature to the critical demixing temperature.^18,64,167^ Thermophoresis in bulk liquid yields comparably low efficiencies of ≈10^–16^ to 10^–14^,^63,95,168^ while similar active particles confined at liquid–liquid interfaces propelled via the Marangoni effect demonstrate a drastic increase in efficiency of several orders of magnitudes up to ≈10^–11^.^67,169^ Even higher efficiencies are reported for particles propelled by self-electrophoresis (≈10^–9^ to 10^–8^),^61,170^ or self-diffusophoresis (≈10^–9^).^57^ This comparably high efficiency can be explained by additional energy contributions that are not accounted for. Indeed, while our definition of η allows us to roughly estimate the conversion efficiencies and to qualitatively compare the different propulsion mechanisms, we should note that here we neglect any contributions from other energy sources apart from light. These include for example, energy released by consuming chemical fuels such as H_2_O_2_,^61^ energy stored in critical mixtures,^18,64,167^ or energy released by the partial degradation of active particles.^57^ Overall, the light-to-work efficiency for different propulsion mechanisms spans over 7 orders of magnitude. Such a large range highlights how efficiency has not been a design parameter in the development of light-driven active particles, but improvements in efficiency for the various mechanisms are still possible and needed in order to develop real-life functional materials and devices based on light-activated active matter systems.

## Conclusions

In this Perspective, we have discussed the ongoing progress toward actuating and controlling soft active matter by exploiting the different properties of light. While changing the light intensity provides a remote effortless means to adjust the speed and direction of light-activated particles, the potential of other properties of light to control soft active matter actuation has been mostly left untapped. For example, selectivity to light wavelength can enable multiple propulsion mechanisms to coexist on a single particle, e.g., by triggering exclusive light-matter interactions at different sites of the particle.^19,117−119^ Furthermore, light polarization and the transfer of its linear and spin angular momentum can enable complex combinations of translation and rotation in the propulsion of active particles without the need for any additional fuel source.^21,148,149^ Finally, the use of spatiotemporally structured light can combine all of light’s different properties into one powerful tool to control the actuation of active matter systems by light in a way that is flexible, selective and adaptive yet concomitantly easy to operate. Such a level of control through light can enable active matter researchers to test theory (e.g., phase transitions, optimal navigation strategies) as well as to develop applications in energy conversion, catalysis, drug delivery, and tissue engineering taking advantage of the fact that light is a widely available and broadly biocompatible source of energy. Some microorganisms, however, experience phototoxicity^171,172^ which depends on the wavelength (typically UV light produces more damages than visible light, which in turn is more damaging than near-IR light) and intensity (typically damages occur only above a certain intensity threshold and increase for higher intensities) of the illuminating light. However, other light properties (e.g., polarization, angular momentum) can also contribute to phototoxicity, at least in organisms that are sensitive to these properties; nevertheless, their phototoxicity has not been explored until now. To summarize, light offers a diverse range of mechanisms and strategies to control the actuation of active matter. We envisage that exploiting all of its properties will be key to designing responsive and interactive active particle systems with a high level of control to advance the field of soft active matter.
